# Bayes Syndrome and Myocardial Fibrosis: Mechanistic Links and Clinical Implications

**DOI:** 10.31083/RCM47928

**Published:** 2026-06-26

**Authors:** Faxue Li, Maomao Zhao, Rongbo Yu, Xuan Guo, Wenyi Ji, Wenliang Zhang, Qiang Wu

**Affiliations:** ^1^Department of Cardiology, Lanzhou University Second Hospital, 730030 Lanzhou, Gansu, China; ^2^Department of Cardiology, The First School of Clinical Medicine, Lanzhou University, 730013 Lanzhou, Gansu, China; ^3^Department of Neurology, The Second People’s Hospital of Baiyin City, 730910 Baiyin, Gansu, China

**Keywords:** Bayes syndrome, myocardial fibrosis, interatrial block, atrial fibrillation, stroke

## Abstract

Bayes syndrome is characterized by advanced interatrial block (A-IAB), which is associated with atrial fibrillation (AF) and ischemic stroke, and has gained widespread attention in recent years. Myocardial fibrosis, particularly atrial fibrosis, is a crucial substrate for the onset of AF and for atrial electricomechanical dysfunction. Growing evidence indicates a bidirectional relationship between Bayes syndrome and myocardial fibrosis. Myocardial fibrosis leads to delayed atrial conduction and the development of interatrial block (IAB), whereas IAB may exacerbate fibrosis through electrophysiological and hemodynamic changes. Several clinical studies have demonstrated that Bayes syndrome independently predicts the occurrence and recurrence of AF, ischemic stroke, and mortality. Imaging modalities, including late gadolinium enhancement cardiac magnetic resonance (LGE-CMR) and echocardiography, enable noninvasive assessment of myocardial fibrotic burden and atrial structural and functional remodeling. Integrating electrocardiographic markers with imaging phenotypes may improve risk stratification, rhythm-monitoring strategies, and clinical decision-making. However, current evidence remains limited in terms of mechanistic elucidation and prospective clinical validation. This review synthesizes mechanistic and imaging evidence linking Bayes syndrome with fibrosis, discusses the clinical implications, and highlights potential targets for anti-fibrotic strategies and stroke prevention. This review also outlines key knowledge gaps and priorities for future longitudinal studies.

## 1. Introduction

Bayes syndrome is a clinical condition characterized by advanced interatrial block (A-IAB), frequently associated with atrial fibrillation (AF) and ischemic stroke [1]. The earliest account of this condition dates back to Bachmann’s description of interatrial block in 1941 [2]. Starting in 1979, Bayés de Luna and colleagues systematically defined the unique anatomical-electrical entity of “interatrial block” classifying it into partial and complete types, and uncovering the link between A-IAB and supraventricular arrhythmias, thereby establishing a distinct arrhythmic syndrome—Bayes syndrome [3,4]. From an anatomical standpoint, interatrial electrical conduction predominantly depends on pathways such as the Bachmann bundle [5,6]. When these pathways are structurally or functionally impaired due to myocardial fibrosis, ischemia, or degenerative changes, conduction delay or interruption may occur, manifesting on the electrocardiography (ECG) as prolonged P-wave duration (≥120 ms) and characteristic changes, such as bidirectional P-waves in the inferior leads [3,7,8]. Myocardial fibrosis, characterized by excessive deposition of extracellular matrix, destruction of gap junctions, and increased conduction heterogeneity, serves as a common anatomical substrate for IAB and reentrant arrhythmias, such as atrial AF [9,10]. Atrial asynchrony caused by IAB, along with hemodynamic disturbances, may further exacerbate atrial wall stress and the fibrotic response, leading to the formation of an “electrical-structural positive feedback” loop [7]. Despite extensive electrophysiological research on Bayes syndrome since its introduction by Bayés de Luna in 1979, there remains a lack of systematic summaries regarding its structural changes, fibrosis distribution patterns, and molecular mechanisms, with clinical practice primarily focused on ECG findings and AF risk assessment.

In recent years, with advancements in imaging techniques such as transthoracic echocardiography (TTE) and cardiac magnetic resonance (CMR), researchers have gained the ability to directly assess atrial fibrosis, a key pathological change, offering new perspectives to further elucidate its mechanisms at both the tissue and functional levels [11,12]. Consequently, this review seeks to systematically clarify the central role of myocardial fibrosis in the onset and progression of Bayes syndrome, emphasizing the bidirectional pathological and physiological relationship between fibrosis and atrial conduction block. It further investigates the predictive and stratification value of this mechanism in relation to atrial fibrillation and stroke risk, offering a theoretical foundation for personalized treatment and targeted anti-fibrosis strategies.

## 2. Bayes Syndrome

### 2.1 Definition and Pathological Basis

Bayes syndrome, also known as interatrial block syndrome, is defined as a clinical condition characterized by electrocardiographic evidence of IAB, which is frequently associated with supraventricular tachyarrhythmias (especially atrial fibrillation and atrial flutter), left atrial dysfunction, and serious complications such as ischemic stroke [1]. Since the initial report of A-IAB in 1956, this syndrome has progressively gained recognition among clinical researchers [13]. In 1979, Bayés de Luna and colleagues provided the first systematic description of the concept of “interatrial block,” establishing a clear distinction from intra-atrial block and proposing a classification into partial (incomplete) and complete block ( A-IAB), thereby laying the foundational definition for Bayes syndrome [4,14]. Subsequent research in 1988 confirmed that patients with A-IAB exhibited a higher propensity for developing supraventricular arrhythmias compared to those with partial IAB, suggesting a central role for A-IAB in atrial electrophysiological dysfunction [15,16].

There are significant individual anatomical variations in the interatrial electrical conduction pathways in humans, which can be categorized into three main types: (1) the Bachmann’s bundle, serving as the principal forward-conducting pathway; (2) atrial muscle bridges traversing the level of the right pulmonary veins, also referred to as the foramen ovale region connection; and (3) conduction pathways located within and around the coronary sinus ostium [5,6,11,17]. These structures function collaboratively to maintain electrical synchrony between the right atrium (RA) and the left atrium (LA). Structural or functional impairment of any of these pathways can lead to interatrial conduction delay or block, which is manifested electrocardiographically by prolonged P-wave duration or abnormal waveform morphology [7]. It is noteworthy that there is no well-defined “bundle-like” structure for conduction between the sinoatrial node and the atrioventricular node; rather, the conduction mainly proceeds through the superior atrial regions. As the primary interatrial conduit, Bachmann’s bundle originates from the anterior wall of the RA, crosses the interatrial septum, and terminates at the anterior wall of the LA; its anatomical and functional integrity is crucial for maintaining synchronous atrial contraction [16]. Studies have indicated that disruption or fibrotic change in the continuity of the circular myocardial fibers in this region represents the key anatomical substrate for interatrial conduction block [5].

### 2.2 Clinical Manifestations

#### 2.2.1 Electrocardiographic Pathophysiology and Manifestations of Bayes Syndrome

During normal sinus rhythm, the primary anatomical pathway for interatrial conduction is Bachmann’s bundle. Conduction delay or complete block within this pathway can result in A-IAB. In addition, the conduction structures located in the coronary sinus, the anterior superior septum, and the posterior inferior septum can also electrically connect the two atria [18]. In A-IAB, conduction through Bachmann’s bundle is functionally or anatomically “blocked”, and the sinus impulse is often redirected through the coronary sinus (and, less commonly, via the fossa ovalis region) to activate the left atrium in a caudo-cranial (tail-to-head) direction. The corresponding electrocardiographic manifestation is a biphasic P wave (+/−) in the inferior leads (II, III, aVF), indicating dissociation of the depolarization sequence between the left and right atria and electromechanical asynchrony [8,19]. The morphology and duration of the atrial P wave reflect the depolarization process and timing of the left and right atria. Delayed interatrial electrical conduction leads to alterations in P-wave duration and morphology [20]. Based on P-wave characteristics, IAB is classified into the following categories:

A. A normal P-wave duration is defined as less than 110 ms by the World Health Organization/International Society and Federation of Cardiology Working Group; a duration exceeding 110 ms suggests interatrial block. However, Bayes de Luna et al. [1,8,21] have proposed a P-wave duration of ≥120 ms for the diagnosis of interatrial block.

B. The presence of biphasic P waves and dome-shaped, peaked P waves [16,18].

C. IAB results from delayed or blocked atrial conduction, producing a broad P wave (≥110 ms), typically bifid. This indicates an electrical delay in left-right atrial activation. With progression, the sinoatrial impulse may retrogradely activate the left atrium via inferior pathways (e.g., coronary sinus), resulting in biphasic P waves in the inferior leads [22]. In 2025, Wang and Gu [23] reported a case of a 71-year-old woman with a history of paroxysmal atrial fibrillation. Before sinus rhythm ablation, her ECG showed a P wave duration of approximately 160 ms and biphasic P waves in leads II, III, and aVF, indicating the presence of IAB.

D. The angle between the two limbs of the biphasic P wave is greater than 120°.

In a 2012 publication, Bayes de Luna et al. [8] emphasized that three criteria must be satisfied to define ECG patterns resulting from conduction block or delay, all of which are met in the case of interatrial block (IAB):

(1) The ECG pattern may appear intermittently or transiently and is independent of heart rate changes;

(2) The ECG manifestation can occur in the absence of left atrial enlargement;

(3) Its characteristic ECG pattern can be reproduced experimentally.

#### 2.2.2 Clinical Relevance of Bayes Syndrome

In clinical practice, IAB is relatively common and is associated with an elevated risk of atrial fibrillation and increased all-cause and cardiovascular mortality. Although less common, A-IAB is a strong marker for left atrial enlargement (LAE) and paroxysmal supraventricular tachyarrhythmias (including AF) and carries greater prognostic significance [11]. Thus, IAB plays a significant role in predicting the onset and progression of atrial fibrillation.

#### 2.2.3 Pathophysiological Links Between IAB, Atrial Fibrillation, and Stroke

The association between A-IAB and atrial tachyarrhythmias, predominantly AF, is termed Bayes syndrome [24]. A-IAB and AF share common pathophysiological mechanisms and are closely related to atrial fibrosis [25]. Furthermore, a bidirectional and mutually reinforcing relationship exists between them. Specifically, AF induces atrial remodeling and exacerbates fibrosis, thereby promoting the development of IAB. Conversely, IAB triggers electrical reentry in the Bachmann bundle region and generates atrial premature beats, facilitating the onset of AF [7,26]. A-IAB has been established as an independent predictor of AF across various clinical contexts, such as heart failure, cavotricuspid isthmus ablation, pulmonary vein isolation, cardiac amyloidosis, transcatheter aortic valve replacement (TAVR), and electrical or pharmacological cardioversion [27,28,29,30]. Moreover, IAB is associated with left atrial thrombus formation and an increased risk of stroke even during sinus rhythm, potentially through several mechanisms. IAB may promote thrombosis by causing slow blood flow due to electromechanical dissociation, increasing prothrombotic tendency associated with underlying ﬁbrotic atrial cardiomyopathy (FACM), and inducing atrial conduction abnormalities. A prospective international cohort study enrolled 556 subjects aged 70–100 years without baseline AF. During 23 months of follow-up, A-IAB was significantly associated with incident AF (hazard ratio [HR] 2.9, 95% CI 1.7–5.1, *p* < 0.001) and stroke (HR 3.8, 95% CI 1.4–10.7, *p* = 0.010) [31]. Another combined analysis based on the Atherosclerosis Risk in Communities (ARIC) and Multi-Ethnic Study of Atherosclerosis (MESA) community cohorts included over 2900 patients to evaluate the incremental value of incorporating P-wave indices (prolonged P-wave duration, A-IAB, abnormal P-wave axis, abnormal terminal force in lead V1) into the CHA_2_DS_2_-VASc score. The results demonstrated that both abnormal P-wave axis and A-IAB were independently associated with AF-related ischemic stroke, suggesting that P-wave indices could serve as a valuable supplement to traditional scoring systems for guiding anticoagulation therapy [32]. Therefore, through its bidirectional relationship with atrial fibrosis and AF, A-IAB serves as an important independent predictor of AF and stroke risk and holds promise for optimizing clinical risk stratification and anticoagulation strategies.

## 3. Myocardial Fibrosis

### 3.1 Correlation Between Myocardial Fibrosis and IAB

Histological evidence demonstrates that fibrotic FACM serves as a common anatomical substrate for AF and A-IAB [25]. Both AF and A-IAB have been shown to induce atrial remodeling, increase the fibrotic burden, and potentially initiate thrombotic cascade reactions [33]. Multiple factors, including microvascular ischemia, heart failure, and infiltrative cardiomyopathies, can contribute to myocardial fibrosis in the atria and Bachmann’s bundle region [26]. As the disease progresses, fibrotic tissue demonstrates increased heterogeneity and disorganized neural distribution, which disrupts intercellular coupling and enhances tissue anisotropy, thereby slowing or even blocking atrial conduction [9,10]. These structural and functional alterations markedly impair the electromechanical coordination of the heart, providing the pathological foundation for the development of IAB. The following sections will elaborate further on the types of fibrosis closely associated with IAB and their underlying pathogenic mechanisms.

#### 3.1.1 Pathological Mechanisms of Myocardial Fibrosis

##### 3.1.1.1 Activation and Proliferation of Fibroblasts

Myocardial fibrosis is primarily driven by the activation and proliferation of cardiac fibroblasts (CFs) [34]. CFs are widely distributed throughout the myocardial interstitium, epicardium, and perivascular regions, and they serve as the principal effector cells in the fibrotic response [35].

(i) Following acute myocardial injury, pro-inflammatory cytokines are upregulated, leading to immune cell infiltration; inflammation and mechanical stress together promote CF activation and transdifferentiation into a myofibroblast phenotype, which is characterized by the expression of alpha-smooth muscle actin (α-SMA) [36].

(ii) Activated CFs secrete large amounts of collagen and other extracellular matrix (ECM) proteins to maintain cardiac structure and function [37]. However, excessive collagen deposition reduces ventricular compliance, exacerbates heart failure (HF), and disrupts the electromechanical coupling of cardiomyocytes, thereby increasing the risk of arrhythmias and mortality [38].

(iii) Perivascular inflammation and fibrosis can impair microcirculatory perfusion, reduce oxygen and nutrient supply, and promote fibrosis toward an irreversible stage [39].

Overall, the “inflammation/mechanical stress–CF activation–ECM overdeposition” cascade represents the key pathological mechanism underlying the onset and progression of myocardial fibrosis, thereby forming the structural basis for subsequent electromechanical dyssynchrony associated with IAB.

##### 3.1.1.2 Inflammatory Response

After myocardial injury, the inflammatory response is considered a critical component of the repair process [40]. Similar types of injury (such as myocardial infarction, infection, or toxic damage) can all activate inflammatory pathways [41], leading to infiltration of inflammatory cells (such as macrophages, monocytes, mast cells, and lymphocytes) into the myocardial tissue [42]. These cells secrete cytokines, including tumor necrosis factor-alpha (TNF-α) and interleukin-1beta (IL-1β), promoting fibroblast proliferation and their transformation into a myofibroblast phenotype, thereby driving scar formation [40,43]. Moderate inflammatory fibrosis during the acute phase contributes to the stabilization and repair of the injured area; however, if the response is excessive or persistent, it may result in myocardial stiffness, reduced compliance, and impaired contractile function, ultimately exacerbating heart failure [44]. Therefore, inflammatory myocardial fibrosis plays a dual role in repair and adverse remodeling, and its precise regulation is essential for improving outcomes after myocardial infarction [40].

##### 3.1.1.3 Oxidative Stress

Oxidative stress induces the excessive production of reactive oxygen species (ROS) and reactive nitrogen species (RNS), leading to damage of critical cellular components including proteins, lipids, and DNA, and thereby disrupting cellular homeostasis.

(i) Basal levels of ROS are essential for physiological processes including cell signaling, microbial defense, gene expression, cell growth, and programmed cell death, persistent oxidative stress activates apoptotic pathways, resulting in cardiomyocyte death [38].

(ii) ROS, as key signaling molecules, can activate pathways such as transforming growth factor-beta (TGF-β)/Smad, which promotes the transdifferentiation of cardiac fibroblasts into myofibroblast phenotypes and is accompanied by excessive deposition of collagen and other extracellular matrix components, thereby facilitating the progression of myocardial fibrosis [45].

(iii) Oxidative stress can activate signaling pathways such as nuclear factor kappaB (NF-κB), upregulating the expression of various inflammatory factors, including TNF-α and IL-1β, which amplifies the inflammation-fibrosis positive feedback loop and further exacerbates tissue remodeling and functional impairment [46].

##### 3.1.1.4 Mechanical Load and Pressure Overload

**Fig. 1.**
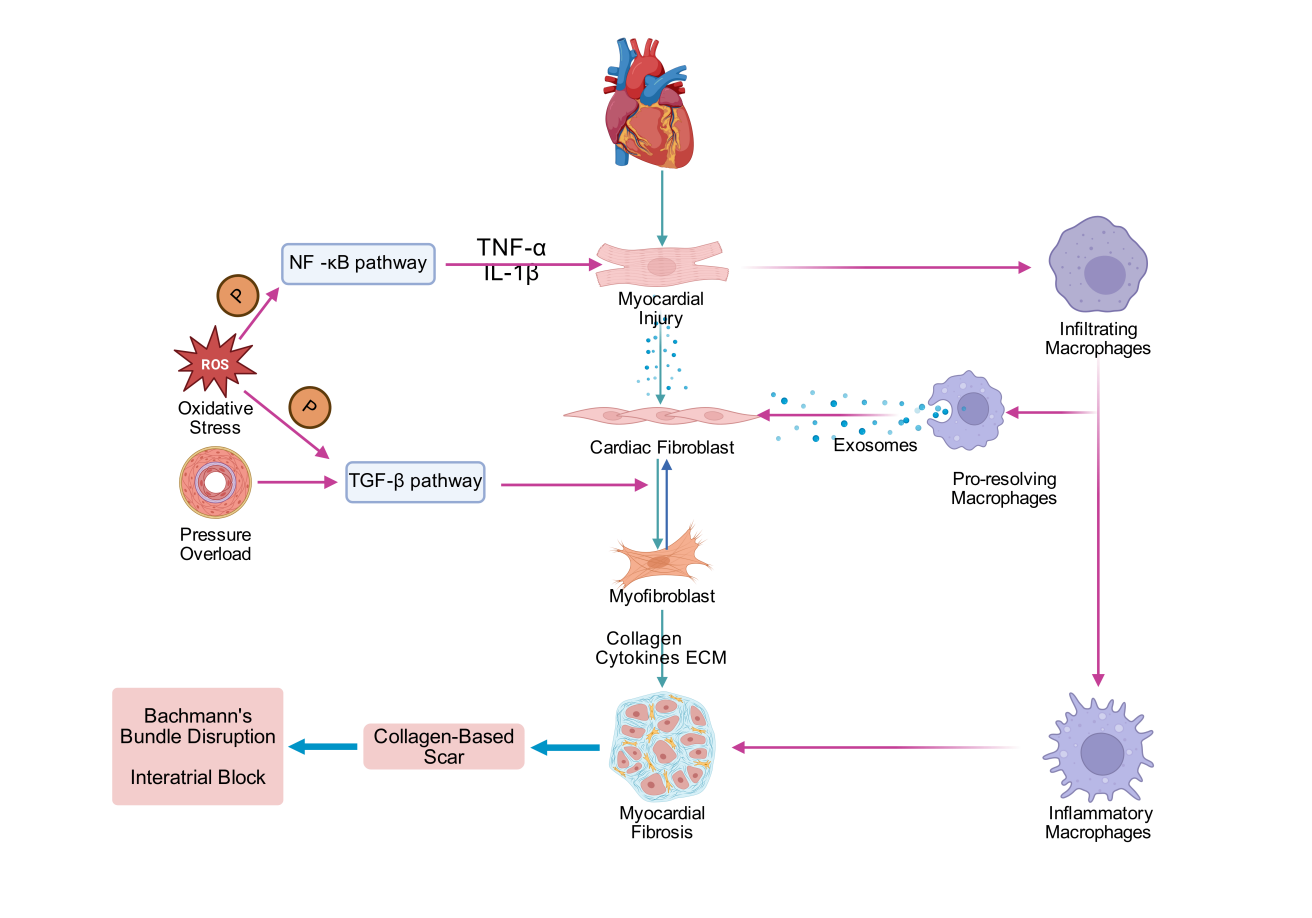
**The pathophysiological mechanisms underlying myocardial fibrosis and the subsequent pathway leading to IAB**. This schematic provides a guided overview of the proposed pathway linking pressure overload/oxidative stress to inflammatory and pro-fibrotic remodeling. Pressure overload and oxidative stress activate NF-κB and TGF-β signaling, amplifying inflammatory cytokine production. Following myocardial injury, macrophage infiltration and phenotypic transitions enhance cytokine- and exosome-mediated communication with cardiac (atrial) fibroblasts, promoting myofibroblast differentiation. The resulting collagen/ECM accumulation drives fibrosis and collagen-based scar formation, which may impair interatrial conduction (including Bachmann’s bundle) and contribute to IAB. IAB, advanced interatrial block; TGF-β,transforming growth factor-β; NF-κB, nuclear factor kappaB; ECM, extracellular matrix; ROS, reactive oxygen species; TNF-α, tumor necrosis factor-alpha; IL-1β, interleukin-1beta. Figure created using Scientific Image and Illustration Software | BioRender.

Chronic pressure overload (e.g., hypertension or aortic stenosis) initially induces compensatory myocardial hypertrophy to restore wall stress and maintain pump function [47,48]. However, when hypertrophy progresses beyond the perfusion supply threshold, myocardial ischemia, necrosis, and interstitial collagen deposition occur. This leads to fibrosis accumulation, a transition from compensation to decompensation, and ultimately results in heart failure and an increase in adverse events [49]. Mechanistically, angiotensin II drives pressure overload–associated myocardial interstitial fibrosis by activating the TGF-β signaling axis, promoting fibroblast activation and excessive extracellular matrix deposition [50]. Clinically, in hypertensive heart disease (HHD), the fibrotic burden serves as an independent prognostic marker, its severity being positively correlated with reduced myocardial compliance, an increased risk of heart failure hospitalization, and mortality. This highlights the translational importance of early identification and intervention for pressure overload–induced fibrosis [51] (Fig. 1; Table 1, Ref. [5,11,39,51,52,53,54,55]).

**Table 1.** **Relationship between myocardial fibrosis and interatrial block (IAB)**.

Type of fibrosis	Main etiologies	Core pathological mechanisms	Pathological & electrophysiological consequences	Relationship with IAB	References
Replacement (Reparative) fibrosis	MI, myocarditis, infection, toxic injury, I/R	Inflammatory response: cytokines from inflammatory cells promote scar formation	Well-defined fibrotic scars replacing necrotic cardiomyocytes	Scar tissue forms a physical conduction barrier, directly interrupting intra-atrial conduction pathways (Bachmann’s bundle), serving as the anatomical basis for IAB.	[5,11,52]
		Oxidative stress: ROS mediates cardiomyocyte injury and fibrotic signaling			
Interstitial (Reactive) fibrosis	Pressure overload (hypertension, aortic stenosis), aging, metabolic syndrome, cardiomyopathy	Mechanical stress: pressure overload activates TGF-β pathway, promoting ECM synthesis	Diffuse collagen deposition in interstitium and perivascular regions, myocardial stiffening, reduced tissue homogeneity	Collagen deposition increases electrical impedance, slows conduction velocity, and enhances tissue anisotropy, contributing to conduction delay (IAB).	[39,51,54]
		Sustained fibroblast activation: CFs activated without necrosis secrete collagen			
Mixed fibrosis	Chronic myocardial injury (ischemic heart disease, HF, infiltrative diseases)	Oxidative stress: ROS induces apoptosis and enhances ECM synthesis	Combination of reparative scars and diffuse interstitial collagen network	Combines conduction blockade from replacement fibrosis and conduction slowing from interstitial fibrosis, synergistically increasing IAB risk and severity.	[53,55]
		Inflammatory response: pro-inflammatory cytokines activate CFs without necrosis			

IAB, interatrial block; MI, myocardial infarction; HF, heart failure; I/R, ischaemia/reperfusion; ROS, reactive oxygen species; TGF-β, the transforming growth factor-β; ECM, extracellular matrix; CFs, cardiac fibroblasts.

#### 3.1.2 Classification of Myocardial Fibrosis

Cardiac fibrosis is broadly classified into replacement fibrosis and interstitial fibrosis, which exhibit significant differences in their pathophysiological mechanisms and clinical implications. Replacement fibrosis commonly occurs following cardiomyocyte necrosis caused by ischemia, ischemia/reperfusion, inflammation, or toxic injury, where fibrous tissue replaces the dead myocardium. It is considered a typical form of reparative fibrosis, reflecting the scar healing process following tissue injury [52]. In contrast, interstitial fibrosis does not require extensive cardiomyocyte loss but is characterized by collagen deposition and expansion within the myocardial interstitium and perivascular regions. It is often observed under conditions such as chronic pressure or volume overload, recurrent transient ischemia, aging, and cardiomyopathies, and is categorized as reactive fibrosis [53]. In summary, these two types of fibrosis correspond to acute necrotic repair and chronic stress responses, respectively. Their distinct pathological backgrounds and clinical outcomes indicate that fibrosis resulting from different etiologies should be addressed differently, and that preventive and therapeutic strategies require individualization.

### 3.2 Imaging Features

Using interatrial septal thickness as an imaging surrogate for IAB to predict recurrence risk after catheter ablation of paroxysmal atrial fibrillation has been proven to be ineffective [11]. Pathologically, IAB-related fibrosis is not confined to the Bachmann’s bundle (BB) region but is more accurately represented by diffuse remodeling of the left atrial free wall. Therefore, the focus of phenotypic assessment should be shifted from “local thickness” to “overall atrial fibrosis burden and distribution”. Specifically, left atrial volume and strain phenotypes can be evaluated using TTE, combined with the CMR-late gadolinium enhancement (LGE) technique and T1/T2 mapping to quantify the degree and spatial heterogeneity of fibrosis. This approach enables more accurate characterization and risk stratification of IAB and Bayes syndrome [12]. Nevertheless, several practical limitations of fibrosis imaging should be acknowledged when translating these modalities into routine practice. Late gadolinium enhancement cardiac magnetic resonance (LGE-CMR) provides a relatively direct, noninvasive characterization of atrial fibrosis; however, its routine clinical implementation is constrained by limited availability and cost, the time demands of acquisition and post-processing, and contrast-related concerns in selected patients (e.g., those with renal impairment). Moreover, because of the thin atrial wall and heterogeneity in segmentation threshold selection and post-processing workflows, inter-center reproducibility and standardization remain suboptimal [56]. In contrast, echocardiography is more readily accessible, yet speckle-tracking echocardiography (STE)-derived left atrial strain/strain rate is sensitive to image quality, loading conditions, rhythm irregularity, and vendor-/software-specific algorithms, resulting in substantial inter-laboratory measurement variability [57].

#### 3.2.1 Cardiac Magnetic Resonance (CMR)

CMR is widely used in clinical practice and research due to its non-invasive nature, multiparametric imaging capability, and high resolution. It enables detailed visualization of cardiac structures, assessment of cardiac function and hemodynamics, and identification of pathophysiological changes including edema, inflammation, microvascular obstruction (MVO), myocardial necrosis, and fibrosis [58,59]. FACM often constitutes the anatomical substrate for AF and A-IAB. LGE with gadolinium-based contrast agents (GBCAs) is the preferred non-invasive technique for detecting FACM. This technique is based on the differential wash-in/wash-out kinetics of the contrast agent between normal myocardium and fibrotic tissue, which causes fibrotic regions to appear as delayed hyperintense signals approximately 10 minutes after intravenous injection, thereby enabling clear visualization and quantitative assessment of fibrosis [40,41,42]. In recent years, the application of three-dimensional reconstruction techniques in CMR has further enhanced the spatial resolution for visualizing atrial fibrosis in IAB patients. This advancement is particularly useful for direct visualization of lesions in the Bachmann’s bundle region, providing robust evidence for elucidating the anatomical basis of IAB [11]. Beyond fibrosis assessment, CMR also holds significant clinical value for predicting the risk of atrial fibrillation recurrence after catheter ablation. Studies have shown that both extensive atrial fibrosis and the deposition of enhanced scar tissue post-ablation are associated with lower recurrence rates [60]. Furthermore, similar to TEE, CMR can also identify fibrotic lesions at the level of the left atrium. These fibrotic regions are not only closely associated with interatrial conduction delay but are also indicative of an increased future risk of IAB and supraventricular arrhythmias in patients [12].

#### 3.2.2 Echocardiography

**Fig. 2.**
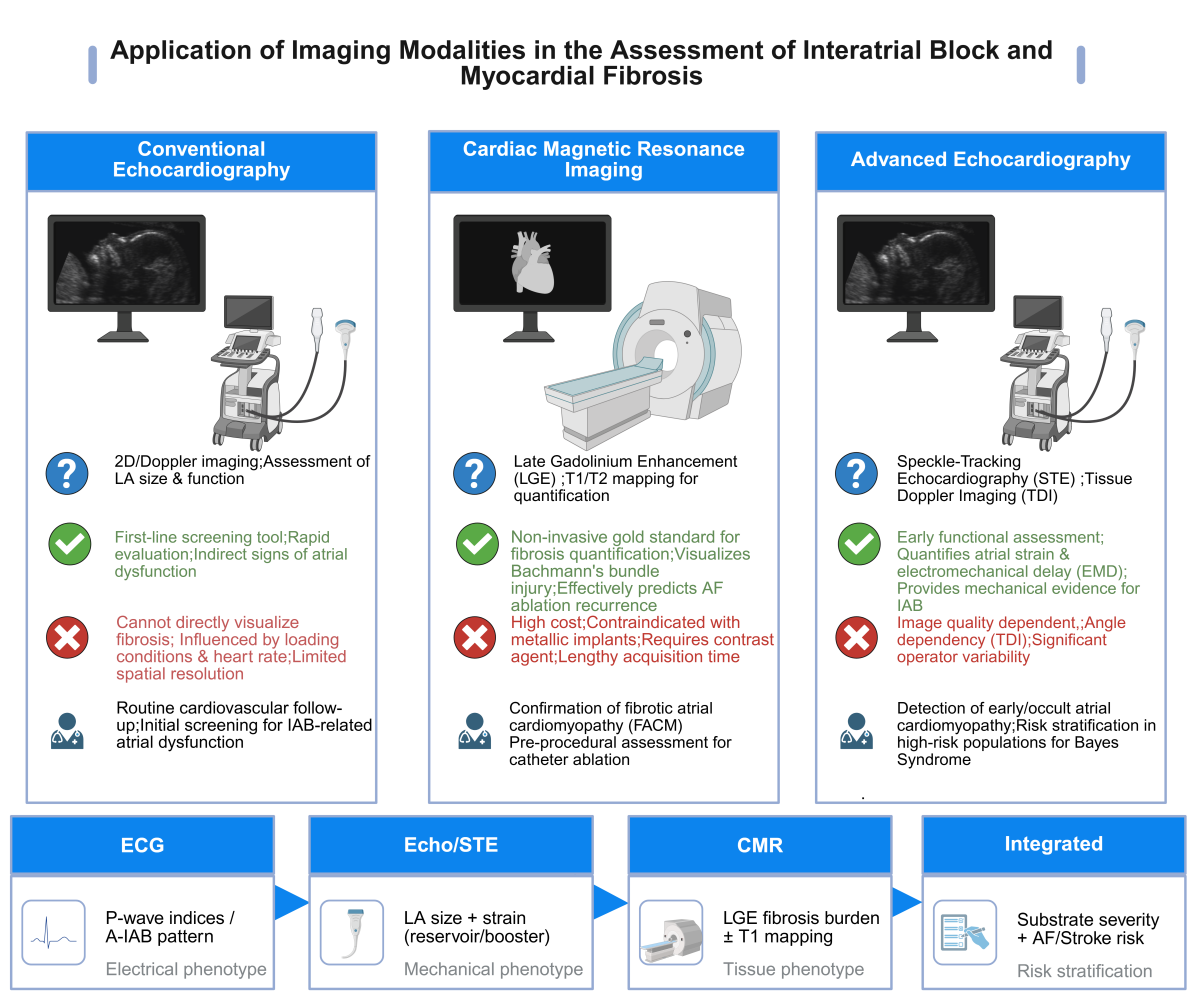
**Application of imaging modalities in the assessment of interatrial block and myocardial fibrosis**. The upper panels summarize key imaging approaches—conventional echocardiography, CMR and advanced echocardiography (STE and TDI)—highlighting their major readouts, advantages, limitations, and typical clinical applications in evaluating atrial structure/function and fibrosis-related phenotypes. The lower schematic illustrates the complementary, stepwise strategy for integrated phenotyping and risk stratification: ECG (electrical phenotype; P-wave indices/A-IAB pattern) → echo/STE (mechanical phenotype; LA size and strain) → CMR (tissue phenotype; LGE fibrosis burden ± T1 mapping) → integrated assessment to estimate substrate severity and AF/stroke risk. LA, left atrium; AF, atrial fibrillation; IAB, interatrial block; FACM, fibrotic atrial cardiomyopathy; STE, speckle-tracking echocardiography; CMR, cardiac magnetic resonance; TDI, tissue Doppler imaging; EMD, electromechanical delay; ECG, electrocardiogram; LGE, late gadolinium enhancement. Figure created using Scientific Image and Illustration Software | BioRender.

The size of the LA has been identified as an important predictor of cardiovascular morbidity and mortality [61]. Early tissue Doppler imaging (TDI), used for assessing atrial function, was limited by poor reproducibility and restricted clinical application due to angle dependence, numerous signal artifacts, and sampling geometry constraints, which made it difficult to accurately characterize motion in thin-walled, high-curvature regions such as the atrial appendage apex [62]. In contrast, speckle-tracking technology can overcome these limitations, enable quantification of myocardial function in relevant regions, and detect declines in atrial compliance prior to the onset of atrial enlargement [63]. TTE is widely used to assess the anatomical structure and functional status of the LA [64]. With the development of new techniques such as STE, not only can atrial wall deformation be more precisely identified, but functional changes closely associated with atrial fibrosis can also be revealed, which is particularly pronounced in IAB patients [12,65]. The atrial electromechanical delay (EMD) and P-wave dispersion can be quantitatively assessed using TDI [66]. EMD is defined as the interval from the onset of atrial depolarization to the initiation of mechanical contraction, anchored at the onset of the P wave on ECG and measured to the corresponding mechanical event using TTE Doppler or tissue velocity imaging [61]. The latter evaluates conduction heterogeneity via multi-lead ECG, and is used for assessing atrial remodeling and predicting atrial fibrillation risk [67]. The decline in atrial contribution to ventricular filling and ejection in IAB, resulting from atrial fibrosis, can be captured by pulse Doppler through measurement of the peak velocities of the early mitral inflow (E) and atrial filling (A) waves [12]. In cases of extensive fibrosis, LA diastolic function is impaired, and chamber pressure and wall stress increase, which may further drive the fibrotic process; the amplitude of the A wave and its relative contribution to ventricular filling may also change, requiring prospective validation [68]. In summary, an integrated multi-parameter approach utilizing TTE, STE, TDI, and pulse Doppler can comprehensively characterize the impact of atrial fibrosis on atrial structure and function, thereby enhancing the diagnosis, stratification, and management of IAB (Fig. 2).

## 4. The Relationship Between Bayes Syndrome and Myocardial Fibrosis

### 4.1 Pathophysiological Link

Myocardial fibrosis plays a pivotal role in the pathophysiological process of Bayes syndrome. Similar to AF [69], the prevalence of IAB increases with age [20,70], which may be associated with the cumulative degree of myocardial fibrosis [71]. Intra-atrial and inter-atrial conduction is slowed and fragmented by fibrosis through increased extracellular matrix volume fraction, disrupted gap junctions, and enhanced conduction anisotropy, thereby inducing varying degrees of conduction block [38,54,72,73,74,75]. Multimodal imaging, such as LGE-CMR and STE, can noninvasively identify fibrotic phenotypes, aiding in the delineation of the etiological substrate of Bayes syndrome and facilitating risk stratification. In IAB patients, atrial contraction dyssynchrony and delay elevate left atrial pressure, further activating fibrotic pathways, making fibrosis both a trigger and a consequence of IAB progression [7]. Diffuse left atrial fibrosis is common in the IAB population; increased ECM slows conduction, elevates atrial wall stiffness, and leads to diastolic dysfunction, which in turn exacerbates electrical conduction abnormalities and IAB risk [61,71,76]. Accordingly, myocardial fibrosis and IAB may be linked through bidirectional pathophysiological coupling, constituting a working model that warrants further validation. This concept supports integrating multimodality imaging with electrocardiographic/electrophysiological phenotyping and motivates investigation of potential benefits from antifibrotic and cardiac unloading strategies.

### 4.2 Potential Mechanisms Linking Bayes Syndrome to Myocardial Fibrosis

It should be noted that the pathophysiological evidence supporting “IAB-driven myocardial fibrosis” remains limited at present [25]. Current understanding has largely been derived from imaging-based structural phenotype coupling and from extrapolating mechanisms involving hemodynamic loading, inflammation, and neurohumoral activation as described in studies of AF and atrial cardiomyopathy. In rigorously defined populations with A-IAB, longitudinal studies integrating serial imaging, circulating biomarkers, and histological validation are still lacking [11].

#### 4.2.1 Electro-Mechanical Activity Disorder

Myocardial fibrosis is promoted by IAB, which serves as a key mediator in this process. IAB reflects dual abnormalities of left atrial structural remodeling and electromechanical dysfunction, with its core feature being atrial activation asynchrony [70,77]. This asynchrony disrupts coordinated atrial contraction, leading to elevated atrial pressure and dilation, which subsequently activate fibrotic signaling pathways and drive the progression of atrial fibrosis [7]. Moreover, abnormal mechanical load and hemodynamic alterations can induce endothelial dysfunction and inflammatory responses, resulting in local thrombosis and increased risk of cardioembolic events. The potential mechanism involves delayed atrial electrical activation causing mechanical conduction asynchrony, which leads to abnormal atrial pressure fluctuations and hemodynamic disturbances, subsequently triggering endothelial activation and inflammatory responses, thereby continuously promoting myocardial fibrosis [11,78]. As fibrosis progresses, atrial wall stiffness increases and diastolic compliance decreases, further slowing atrial electrical conduction, creating a vicious cycle that accentuates the occurrence and progression of IAB.

#### 4.2.2 Activation of Neurohumoral Factors

The renin-angiotensin-aldosterone system (RAAS) is a critical neuroendocrine and paracrine axis, extensively involved in regulating various physiological processes in the cardiovascular system, lungs, and kidneys [79]. Upon activation of RAAS, the level of angiotensin II (Ang II) increases significantly, serving as a key effector molecule promoting fibrosis [75,80].

(i) Multiple downstream signaling pathways are triggered by the binding of Ang II to its type 1 receptor (AT_1_R), including the G protein-dependent phospholipase C (PLC) pathway, which promotes the generation of inositol trisphosphate (IP_3_) and diacylglycerol (DAG), thereby mediating intracellular Ca^2+^ elevation and inducing activation and proliferation of CFs [78,80,81].

(ii) Ang II can stimulate NADPH oxidase (NOX2) to promote excessive production of ROS, triggering the opening of mitochondrial permeability transition pores (MPT) and generating a ROS amplification cascade [82,83]. Mitochondria-derived ROS further activate the c-src pathway, suppressing the expression of the gap junction protein Cx43, thereby disrupting atrial electrical coupling and accelerating fibrosis progression [84].

(iii) Ang II activates the MAPK signaling axis, inducing the secretion of TGF-β_1_, which in turn upregulates AT_1_R density and connective tissue growth factor (CTGF) expression, forming a pro-fibrotic positive feedback loop [85,86].

IAB may lead to abnormalities in atrial electrical activity, which can subsequently impair atrial mechanical function. Such atrial dysfunction may induce alterations in atrial pressure and volume, and these changes are likely to influence the activation of the renin-angiotensin system (RAS) through neurohumoral mechanisms [87]. Therefore, atrial contraction dyssynchrony induced by IAB may activate the RAAS via neurohumoral pathways, exacerbating atrial remodeling and fibrosis. However, experimental evidence regarding RAAS activation induced by IAB remains limited, and the precise molecular mechanisms are required to be further investigated.

### 4.3 Ischemic Heart Disease–Related Remodeling and Interatrial Block

Electrophysiological abnormalities caused by myocardial fibrosis. Alexander et al. [88] found that the burden of coronary artery disease is significantly associated with the incidence of IAB, suggesting that fibrosis serves as a common final pathway for various cardiac diseases, including ischemia, and may impair atrial conduction and trigger IAB through shared molecular remodeling mechanisms. Histological evidence further confirms that fibrotic atrial cardiomyopathy constitutes the anatomical substrate for A-IAB. Moreover, atrial fibrosis is interrelated with blood flow stasis, which promotes thrombus formation on the left atrial appendage, a phenomenon observed in both atrial fibrillation and A-IAB [16,25]. Although there is currently no consensus on the causal direction between atrial fibrosis and Bayes syndrome, existing data support a pathophysiological coupling between the two: on one hand, fibrosis induced by ischemia and other multifactorial causes can lead to dispersed atrial conduction and block. On the other hand, the electromechanical dyssynchrony and increased left atrial pressure caused by IAB can accelerate interstitial remodeling through the inflammation–fibrosis axis.

### 4.4 Potential Mechanism of Atrial Fibrillation-Induced Fibrosis Leading to Bayes Syndrome

**Fig. 3.**
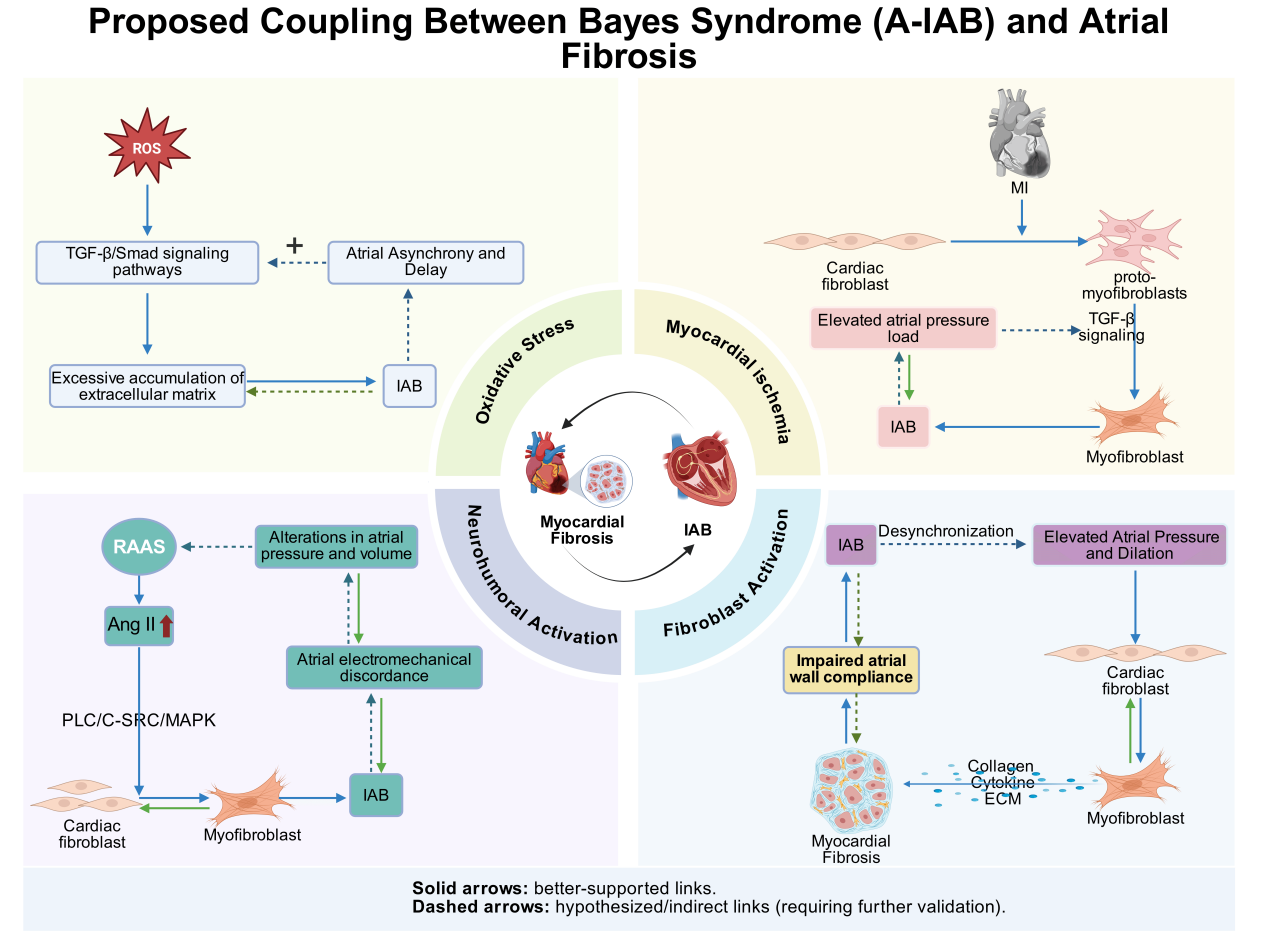
**Proposed coupling between Bayes syndrome (IAB) and atrial fibrosis**. This schematic integrates major mechanistic domains—oxidative stress, ischemic injury, neurohumoral activation (RAAS/Ang II), and mechanical loading—to illustrate a proposed self-reinforcing remodeling loop between atrial fibrosis and A-IAB. The four quadrants organize pathways by domain, and the central loop summarizes the bidirectional coupling: fibrosis facilitates conduction discontinuity and interatrial conduction delay, whereas A-IAB–associated desynchronization and atrial pressure overload may further promote remodeling. Solid arrows denote better-supported links; dashed arrows indicate hypothesized or indirect pathways requiring further validation. ROS, reactive oxygen species; IAB, interatrial block; Ang II, angiotensin II; TGF-β, transforming growth factor β; RAAS, renin-angiotensin-aldosterone system; PLC, phospholipase C. Figure created using Scientific Image and Illustration Software | BioRender.

Atrial structural-functional remodeling (including fibrosis, left atrial enlargement, and persistent mechanical functional alterations) constitutes the fundamental basis and key contributing factor for the development of IAB [11]. Recent intracardiac electrophysiological mapping studies on Bayes syndrome have shown that the Bachmann bundle can have multiple activation pathways, predominantly from right to left; When the conduction system, particularly the Bachmann bundle region, undergoes fibrosis, left atrial electrical activation may be rerouted via inferior septal alternative pathways in a retrograde tail-to-head manner, resulting in a characteristic interatrial dyssynchrony phenotype [23,89,90]. Consequently, atrial fibrosis is recognized as a central feature of arrhythmogenic structural remodeling and can be quantitatively assessed through echocardiography and cardiac magnetic resonance imaging (including LGE and T1/T2 mapping), serving as a histological surrogate for evaluation and risk stratification in IAB/Bayes syndrome patients [91,92]. On electrocardiography, prolonged P-wave duration (≥120 ms) and biphasic P waves in the inferior leads (II, III, aVF) are manifested, indicating high-grade interatrial conduction impairment and rearrangement of atrial activation sequences [1,8,23]. In patients with extensive fibrosis, impaired atrial diastolic function leads to persistently elevated LA pressure, increasing wall tension and creating a mechanically stress-induced positive feedback loop that further promotes fibrotic progression [12]. As fibrosis progresses, the atrial wall gradually loses elasticity, compliance significantly decreases, ultimately resulting in atrial enlargement and structural remodeling [71]. These morphological changes not only impair the mechanical pumping function of the atria but also disrupt the integrity of electrical conduction pathways, slowing the propagation of impulses and increasing spatial heterogeneity of conduction [55]. Animal studies further confirm that atrial fibrosis can lead to delayed electrical signal propagation due to impaired gap junctions between myocytes, markedly increasing the risk of IAB [12]. Therefore, atrial fibrosis, through a pathological cycle of “structural remodeling—conduction abnormality—further fibrosis”, serves not only as a critical pathological basis for IAB but also potentially as a key determinant of its onset and progression (Fig. 3).

### 4.5 Imaging Evidence for Bayes Syndrome/IAB and the Atrial Fibrosis Phenotype

Available direct imaging evidence indicates that, even among individuals without a prior history of AF, IAB is significantly associated with left atrial fibrosis, left atrial size, and LA structural/functional remodeling phenotypes. In a 2024 cohort study by Phrommintikul et al. [93] including 229 adults who underwent CMR, the prevalence of LA fibrosis was higher in patients with IAB than in those without IAB among participants without a history of AF who nevertheless underwent CMR (70% vs. 21.2%; *p* < 0.001). Similarly, in a 2020 study by Ciuffo et al. [71] of 152 patients undergoing preprocedural ECG and CMR assessment before AF catheter ablation, patients with A-IAB had a higher LA minimal volume index (25.7 vs. 19.9 mL/m^2^, *p* = 0.010), a greater LA fibrosis burden (21.9% vs. 13.1%, *p* = 0.020), and a lower LA peak strain rate (0.99 vs. 1.18, *p* = 0.007) than those without IAB, suggesting a more advanced structural–mechanical remodeling phenotype.

Beyond CMR, STE also supports the mechanical phenotype of IAB. Lacalzada-Almeida et al. [94] compared transthoracic echocardiography and STE findings among 21 patients with partial IAB (pIAB), 22 with A-IAB (aIAB), and 56 without IAB, and found that LA volume indexed to body surface area was higher in IAB patients, while STE revealed reduced absolute strain-rate values for atrial booster pump function (SRa) and early reservoir phase (SRs) in the pIAB group versus the non-IAB group, with more pronounced abnormalities in the aIAB group, indicating impaired LA reservoir and contractile function.

Collectively, evidence from CMR and STE consistently supports significant associations of IAB/A-IAB with LA fibrosis, LA enlargement, and impaired mechanical function. However, because most available studies are cross-sectional or retrospective, the directional causal relationship between fibrosis and IAB remains insufficiently established, and prospective longitudinal studies in rigorously defined A-IAB cohorts incorporating serial imaging and circulating biomarkers are needed to clarify the temporal sequence and potential windows for intervention.

### 4.6 Clinical Prognosis and Risk Magnitude of Bayes Syndrome/A-IAB

#### 4.6.1 Major Clinical Endpoints: Atrial Fibrillation and Ischemic Stroke/Thromboembolism

At the level of clinical endpoints, prospective cohorts, systematic reviews, and meta-analyses have consistently shown that Bayes syndrome/A-IAB is associated with increased risks of AF, ischemic stroke, and thromboembolism (TE), and may be useful for risk stratification. In the BAYES registry of elderly patients with structural heart disease who were in sinus rhythm at baseline and had no prior AF, A-IAB was shown to independently predict incident AF and stroke [31]. In the field of systematic evidence synthesis, Prasitlumkum et al. [95] conducted a meta-analysis of 10 studies including 177,249 participants and reported no association between partial IAB and increased ischemic stroke risk, whereas A-IAB was associated with a higher stroke risk (OR ≈ 1.85, 95% CI 1.37–2.50; *p* = 0.001). Nedios et al. [96] likewise supported a robust association between P-wave duration (PWD) and the risk of stroke or thromboembolic events. Collectively, PWD and IAB are consistently associated with elevated risks of AF, ischemic stroke, and thromboembolism and have been identified as independent predictors in multiple studies; however, their predictive performance across different populations and whether corresponding management strategies can improve clinical outcomes requires further validation in prospective, systematic investigations [26].

#### 4.6.2 Structural/Functional Changes and Prognosis: P-wave Phenotypes and Left Atrial Remodeling

P-wave indices (PWIs) may, to some extent, reflect atrial conduction dispersion and electromechanical coupling abnormalities, thereby corresponding to structural–mechanical phenotypes of left atrial remodeling and providing incremental information beyond traditional clinical risk scores. In the ARIC and MESA cohorts, an abnormal P-wave axis—an ECG correlate of LA abnormality—improved prediction of AF-related ischemic stroke, and the P2-CHA2DS2-VASc score outperformed CHA2DS2-VASc in predicting AF-related ischemic stroke [32]. With respect to structural remodeling, PWD was independently and directly associated with LAE in IAB populations, supporting its role as an electrophysiological surrogate of LA enlargement/structural remodeling [20]. Overall, PWIs—particularly PWD and A-IAB—are more likely to mark LA remodeling and to be independently associated with increased risks of AF and TE events [5]; nevertheless, the temporal sequence and potential windows for intervention remain to be clarified by prospective studies incorporating imaging evidence.

#### 4.6.3 Mortality Endpoints and Limitations of Evidence

In addition to arrhythmia-, stroke-, and thromboembolism-related endpoints, studies suggest that P-wave conduction abnormalities may be associated with long-term mortality risk. Analyses from the National Health and Nutrition Examination Survey (NHANES) showed that PWD was significantly associated with higher cardiovascular mortality (HR 1.13 per 1 standard deviation [SD]) and all-cause mortality (HR 1.06 per 1 SD) [97]. However, studies evaluating endpoints such as heart-failure hospitalization in A-IAB/IAB are largely limited to small observational analyses with unstable effect estimates and inconsistent conclusions, and therefore require confirmation in rigorously phenotyped A-IAB cohorts.

## 5. Clinical Prospects for Treatment

### 5.1 Management of Arrhythmias

Anatomical or functional impairment of Bachmann’s bundle can disrupt interatrial conduction pathways, leading to IAB, which is characterized by distinctive P-wave changes [5]. When conduction through Bachmann’s bundle is completely blocked, A-IAB occurs, with a typical electrocardiographic pattern of markedly prolonged P-wave duration and terminal negativity in the inferior leads. This phenotype has been confirmed as an independent predictor of AF recurrence and can be used to guide individualized rhythm or rate control strategies [7,16]. Given the close association between A-IAB and atrial flutter/fibrillation, the role of antiarrhythmic drugs in preventing atrial tachyarrhythmias should be explored, though higher-quality evidence is still needed for validation [98]. The treatment of IAB mainly involves the following aspects.

### 5.2 Anticoagulant Therapy

#### 5.2.1 Oral Anticoagulation (OAC)

Studies have shown that A-IAB is significantly associated with the risk of AF [99]. IAB is often accompanied by LA structural remodeling and functional impairment, and is regarded as a characteristic manifestation of atrial electromechanical imbalance, which can result in abnormal and delayed left atrial electrical activation, thereby promoting blood flow stasis, microthrombus formation, and thrombus development in the left atrial appendage (LAA) [100,101]. Clinically, thromboembolic events are more common in patients with IAB, particularly among those with higher CHA_2_DS_2_-VASc scores. Notably, even in IAB patients without a history of atrial fibrillation, higher CHADS_2_ or CHA_2_DS_2_-VASc scores can still predict the risk of ischemic stroke or transient ischemic attack (TIA) [102]. Martinez-Sellés et al. [103] suggested that in patients with A-IAB and a CHA_2_DS_2_-VASc score ≥2 accompanied by a P-wave duration ≥160 ms, early initiation of anticoagulation therapy should be considered. In addition, Iomini and Baranchuk proposed (2022) [104] that patients with A-IAB should undergo close follow-up to enable early detection of atrial fibrillation and timely initiation of anticoagulation, thereby reducing the incidence of stroke events, lowering morbidity, mortality, and healthcare burden. Recent randomized controlled evidence provides important context for anticoagulation strategies in individuals without AF but with atrial pathology. In a randomized trial enrolling patients with atrial high-rate episodes (AHREs) (NOAH-AFNET 6), Kirchhof et al. [105] found that edoxaban, compared with placebo, did not significantly reduce the composite endpoint of cardiovascular death, stroke, or systemic embolism, and was associated with an increased risk of bleeding. By contrast, the ARTESiA study (device-detected subclinical AF) showed that apixaban, compared with aspirin, reduced the risk of stroke and systemic embolism but increased major bleeding, indicating the need for individualized trade-offs based on net clinical benefit [106]. In addition, in the ARCADIA trial involving patients with cryptogenic stroke and atrial cardiopathy but without AF, apixaban was not superior to aspirin in preventing recurrent stroke [107]. Taken together, although observational studies suggest a prothrombotic phenotype in severe IAB [7,14,102], existing randomized evidence from related AF-surrogate populations indicates that routine anticoagulation in the absence of documented AF remains uncertain and should be individualized according to stroke risk, bleeding risk, and rhythm-monitoring strategies, underscoring the need for prospective studies enrolling rigorously phenotyped IAB cohorts [7,25].

#### 5.2.2 Left Atrial Appendage Occlusion (LAAO)

Percutaneous left atrial appendage occlusion is an important nonpharmacological strategy for stroke prevention in AF and is increasingly used in patients who are unsuitable for long-term OAC or who have a high bleeding risk [108,109]. The 2023 ACC/AHA/ACCP/HRS AF guideline has recommended LAAO as a reasonable alternative to OAC in such patients [109], and the 2024 ESC AF guideline likewise includes LAAO as an optional stroke-prevention pathway in selected circumstances [110]. Meta-analyses of earlier randomized controlled trials (PROTECT-AF and PREVAIL) and of the randomized trial comparing LAAO with direct oral anticoagulants (PRAGUE-17) indicate that, in carefully selected patients with nonvalvular AF, LAAO achieves efficacy comparable to pharmacological antithrombotic therapy for prevention of stroke/thromboembolism and is associated with lower risks of hemorrhagic stroke, cardiovascular mortality, all-cause mortality, and fatal stroke [108,111]. Comparative studies across devices (e.g., AMULET IDE) have also informed device selection and periprocedural management [112]. Notably, Bayes syndrome/A-IAB is frequently accompanied by left atrial enlargement, impaired mechanical function, and a progressive atrial cardiomyopathy phenotype, which may theoretically affect anatomic sizing, device apposition, and the risks of peri-device leak and device-related thrombus after LAAO, thereby underscoring the importance of preprocedural imaging assessment (TEE/CT) and standardized postprocedural follow-up [7,71]. However, outcome data for LAAO stratified by IAB severity (particularly A-IAB) are currently lacking, and prospective studies are therefore warranted to establish the efficacy and safety of LAAO in patients with Bayes syndrome.

### 5.3 Rhythm Control

#### 5.3.1 Antiarrhythmic Drugs

In terms of rhythm control for IAB, the main strategy involves the early use of antiarrhythmic drugs to prevent and reduce subsequent supraventricular arrhythmias, particularly AF and atrial flutter [25]. In an early small-scale prospective study conducted by Bayés de Luna et al. [19], 32 patients with advanced A-IAB were non-randomly enrolled, and half of them received antiarrhythmic drug therapy. After a 10-month follow-up, the incidence of new-onset atrial fibrillation reached as high as 94% in the untreated group, whereas only 25% of patients receiving antiarrhythmic therapy developed atrial fibrillation, which suggests that early pharmacological intervention may significantly reduce the risk of AF [19]. Although these findings are enlightening, the study was limited by its small sample size, lack of randomization and long-term follow-up data; therefore, there is currently insufficient evidence to support prophylactic use of antiarrhythmic drugs as a standard treatment strategy. Large-scale, multicenter, randomized controlled trials are urgently needed to verify whether early rhythm-control interventions confer long-term benefits in patients with IAB and to determine the optimal drug type and timing of intervention, thereby providing more evidence-based support for the precise management of Bayes syndrome. In addition to prophylactic antiarrhythmic drug use during the IAB stage, recent studies increasingly support implementing early rhythm control promptly after AF onset to reduce AF burden, delay disease progression, and potentially improve major cardiovascular outcomes [25,113,114]. Both the 2023 ACC/AHA/ACCP/HRS atrial fibrillation guideline and the 2024 ESC atrial fibrillation guideline emphasize earlier and more structured rhythm-control strategies in appropriately selected populations. Meanwhile, recent systematic reviews and meta-analyses suggest that early rhythm control is associated with lower risks of cardiovascular mortality, heart-failure events, and stroke [115]. Overall, in high-risk phenotypes such as A-IAB, once AF is detected, early rhythm control can be discussed more proactively, although IAB-specific randomized evidence remains limited.

#### 5.3.2 The Role of Catheter Ablation in Bayes Syndrome/A-IAB

Catheter ablation (CA), with pulmonary vein isolation (PVI) as its cornerstone, is a key rhythm-control strategy for AF [110]. In patients with Bayes syndrome/A-IAB who have a substantial comorbidity burden or recurrent AF, CA may be considered when pharmacological therapy is ineffective or not tolerated. However, prospective ablation studies specifically targeting Bayes syndrome or rigorously defined A-IAB populations remain scarce [11,25]. Available evidence largely comes from retrospective/observational analyses of AF ablation cohorts stratified by IAB phenotypes or P-wave parameters [31]. Collectively, these studies suggest that prolonged baseline P-wave duration and/or an A-IAB phenotype indicate a greater burden of atrial substrate disease and are associated with a higher risk of post-ablation AF recurrence, supporting IAB as a readily obtainable ECG-based substrate marker for preprocedural risk stratification and postprocedural follow-up [116]. From a procedural-strategy perspective, a PVI-only approach should remain the foundational strategy [110]. Given that the key pathophysiological substrate of A-IAB is closely related to impaired conduction in the BB region, targeted ablation or “BB modification” strategies have recently been explored. De Martino et al. [117] reported that, in hybrid ablation for long-standing persistent AF, adjunctive BB ablation is feasible and effective in improving maintenance of sinus rhythm without increasing complication rates. Similarly, Sun et al. [118] proposed in a retrospective propensity score–matched cohort that adding BB modification to circumferential PVI (CPVI) was effective for maintaining sinus rhythm in persistent AF, although the evidence remains observational and requires confirmation in prospective randomized studies. Importantly, any additional ablation lesions may increase iatrogenic scar burden, thereby augmenting conduction anisotropy and predisposing to atrial tachycardia. Therefore, “precise and minimally necessary” lesion deployment guided by electroanatomical markers and imaging-defined fibrosis burden is warranted. For example, Moser et al. (2018) [119] reported that, in strategies targeting isolation of septal fibrotic substrate, residual breakthrough conduction often originated from the BB insertion sites, and focal, limited touch-up ablation at these sites represented a novel and feasible technique to achieve complete isolation of left atrial septal fibrosis. Overall, BB-related strategies are still supported mainly by small-sample or observational evidence, and the optimal target population and net clinical benefit remain uncertain, underscoring the need for prospective studies to validate long-term efficacy and safety.

### 5.4 Cardiac Resynchronization Therapy

Cardiac resynchronization therapy (CRT) has been proven to improve symptoms and prognosis in patients with heart failure. Recent studies have further demonstrated that CRT can significantly shorten P-wave duration and reduce the prevalence of IAB, thus providing an important theoretical basis for its application in the treatment of atrial fibrosis [120]. Although the preliminary results are encouraging, they are still insufficient to recommend CRT as a primary therapeutic approach for IAB. Future studies should include systematic mechanistic investigations and prospective clinical trials to clarify the causal relationship and long-term clinical benefits of CRT in improving atrial fibrosis and interatrial conduction, thereby laying a scientific foundation for its precise application in patients with Bayes syndrome.

### 5.5 Management of Underlying Medical Conditions

#### 5.5.1 Atrial Fibrillation

Angiotensin-converting enzyme inhibitors (ACEIs) and angiotensin II type 1 receptor blockers (ARBs) can reduce the incidence of AF and shorten P-wave duration in patients with hypertension and AF [121]. This therapeutic effect is mainly associated with the inhibition of cytokine-mediated remodeling, reduction of pressure and stretch load, and potential delay in IAB progression, as well as suppression of atrial fibrosis [122]. In addition, regular monitoring is essential for the early identification and dynamic tracking of AF induced by IAB.

#### 5.5.2 Fibrotic Atrial Cardiomyopathy (FACM)

**Fig. 4.**
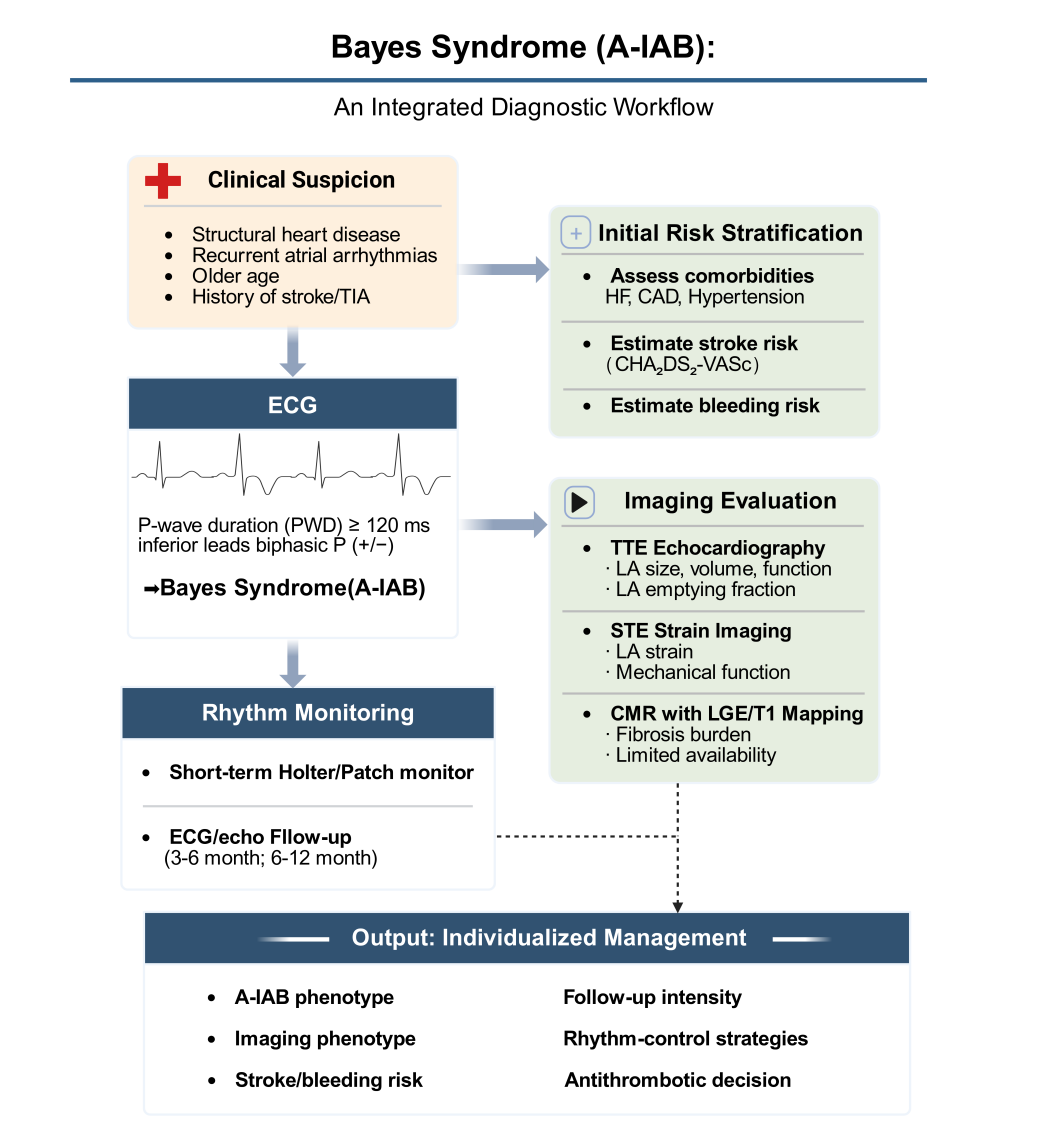
**Integrated diagnostic workflow for Bayes syndrome (advanced interatrial block) and individualized management**. Patients with clinical suspicion undergo 12-lead ECG to identify A-IAB (PWD ≥120 ms with biphasic P waves in inferior leads). Risk stratification and multimodality imaging (TTE ± STE; selective CMR for fibrosis) guide rhythm monitoring and individualized decisions on follow-up intensity, rhythm-control strategies, and antithrombotic management. A-IAB, advanced interatrial block; LA, left atrium; PWD, P-wave duration; TTE, transthoracic echocardiography; STE, speckle-tracking echocardiography; CMR, cardiac magnetic resonance; LGE, late gadolinium enhancement; TIA, transient ischemic attack. Figure created using Scientific Image and Illustration Software | BioRender.

FACM is often the anatomical basis of AF and A-IAB [25], while A-IAB is generally regarded as a precursor of AF onset [94,99]. Therefore, for patients with IAB—especially those at high risk of developing arrhythmias—regular monitoring and follow-up are of significant clinical value. Although cardiac magnetic resonance imaging (C-MRI) combined with LGE is the gold standard for assessing FACM, its use in routine screening is limited by high costs and uncertainty in clinical management strategies [123]. In contrast, ECG and echocardiography (ECHO), as more practical and accessible diagnostic tools, can be effectively used for the dynamic monitoring of IAB. ECG can identify changes in P-wave duration and morphology, thereby detecting the progression of IAB and the occurrence of new adverse events [20,124]. Vectorcardiography (VCG), by analyzing alterations in the P-loop, may more accurately reveal the extent and distribution of atrial fibrosis than surface ECG, particularly when its findings are consistent with fibrosis regions shown by CMR, thus enhancing diagnostic value [8]. Echocardiography can assess left atrial size, function, and atrial wall thickness, indirectly reflecting the extent and severity of atrial fibrosis. Regular examinations not only help clinicians adjust treatment plans in a timely manner but also provide essential prognostic information for patients [1,125,126]. In summary, it is recommended that patients with IAB undergo ECG every 3–6 months and echocardiographic evaluation every 6–12 months to achieve early detection, precise intervention, and long-term risk management [124] (Table 2, Ref. [7,16,19,25,99,100,101,102,103,104,120,121,122,124,125,126]). An integrated diagnostic and follow-up workflow for Bayes syndrome/A-IAB is summarized in Fig. 4.

**Table 2.** **Clinical treatment prospects for Bayes syndrome and myocardial fibrosis**.

Therapeutic domain		Treatment strategy/method	Supporting evidence	Clinical Implications	References
Arrhythmia management		Use of antiarrhythmic drugs to prevent supraventricular tachyarrhythmias	A-IAB predicts AF recurrence. A prospective study showed high AF incidence (94% untreated vs. 25% treated).	May be considered in high-risk patients; higher-quality RCTs needed for standard prophylaxis.	[7,16,19]
Anticoagulant therapy		Anticoagulation for stroke prevention, especially in high-risk patients	A-IAB increases AF and stroke risk. High CHA_2_DS_2_-VASc predicts stroke even in sinus rhythm.	Consider in high-risk IAB patients; close monitoring for AF is recommended to guide therapy.	[99,100,101,102,103,104]
Rhythm control		Early administration of antiarrhythmic drugs to prevent AF and atrial flutter	Antiarrhythmic drugs significantly reduced AF incidence (25% vs. 94%) in a non-randomized study.	Early control may reduce AF risk; evidence insufficient for routine use; large RCTs warranted.	[19,25]
Cardiac resynchronization therapy (CRT)		CRT in patients with heart failure to improve atrial conduction	CRT shortens P-wave duration, reduces IAB prevalence, and improves HF symptoms.	Potential for ameliorating conduction abnormalities; not primary therapy for IAB; more studies needed.	[120]
Management of underlying conditions:	Atrial Fibrillation	Use of ACEIs/ARBs to reduce AF incidence and delay fibrosis	ACEIs/ARBs shorten P-wave duration, reduce AF episodes, and inhibit fibrotic remodeling.	Recommended to attenuate atrial remodeling and IAB progression; regular AF monitoring essential.	[121,122]
	Fibrotic Atrial Cardiomyopathy (FACM)	Regular ECG and echocardiographic monitoring	FACM is the anatomical substrate for AF/A-IAB. ECG and echo detect conduction and structural changes.	ECG every 3–6 months; echocardiography every 6–12 months for dynamic risk stratification and early intervention.	[25,124,125,126]

A-IAB, advanced interatrial block; AF, atrial fibrillation; CRT, cardiac resynchronization therapy; HF, heart failure; ACEI, angiotensin-converting enzyme inhibitors; ARB, angiotensin II type 1 receptor blockers; FACM, fibrotic atrial cardiomyopathy; ECG, electrocardiogram.

#### 5.5.3 Emerging Anti-Fibrotic Therapies and Ongoing Trials

Atrial fibrosis is considered a key structural substrate implicated in the development and progression of Bayes syndrome, and anti-fibrotic strategies have attracted sustained attention in recent years. Pirfenidone, an oral anti-fibrotic agent, has been investigated in populations enriched for myocardial fibrosis (e.g., studies in HFpEF), and has been suggested to reduce myocardial fibrotic burden, thereby supporting anti-fibrotic therapy as a potential treatment direction [127]. Meanwhile, mineralocorticoid receptor antagonists (e.g., spironolactone) may exert upstream modulation of the aldosterone pathway and have also been evaluated in randomized trials within atrial fibrillation cohorts (e.g., IMPRESS-AF); however, existing results indicate inconsistent clinical benefits [128,129], potentially influenced by patient selection and differences in substrate burden. Therefore, direct evidence for anti-fibrotic therapy specifically in rigorously phenotyped IAB populations (particularly A-IAB/Bayes syndrome) remains limited, and future prospective studies integrating imaging-based fibrosis metrics with ECG phenotype stratification are needed to define the appropriate target population and its net clinical benefit.

## 6. Conclusion and Outlook

Bayes syndrome (A-IAB) represents a typical electrocardiographic phenotype of atrial electromechanical coupling abnormalities, with its morphological basis primarily attributed to myocardial fibrosis. The significant association between Bayes syndrome and the risk of atrial fibrillation and stroke has been confirmed by numerous studies; however, the existing evidence is largely derived from observational studies, and the underlying causal mechanisms remain to be elucidated. Future research efforts should be directed toward three levels: (1) Mechanistic exploration—delineating key molecular pathways (e.g., inflammation, oxidative stress, and RAAS/aldosterone signaling) involved in the interplay between fibrosis and interatrial conduction delay, and clarifying their temporal and potentially bidirectional relationships; (2) Clinical validation and risk stratification—establishing a quantitative fibrosis assessment framework in multicenter prospective cohorts by integrating CMR, echocardiographic markers (including left atrial structure/function), and P-wave/A-IAB electrophysiological features, and testing its incremental value for stroke prediction and anticoagulation decision-making beyond conventional clinical scores; (3) Translation and intervention—evaluating upstream therapies targeting atrial remodeling, electrophysiological remodeling strategies, and early rhythm-management approaches in rigorously phenotyped Bayes syndrome populations. Overall, Bayes syndrome is not only a manifestation of atrial conduction abnormality but may also serve as a clinical window into atrial fibrosis and thromboembolic risk. Bidirectional integration of mechanistic insights with prospective clinical validation is essential to strengthen the evidence base for precision diagnosis and management of Bayes syndrome.
